# Editorial: Tuberculosis: recent updates in basic research, drug discovery and treatment

**DOI:** 10.3389/fmed.2025.1668254

**Published:** 2025-08-29

**Authors:** Ameya D. Bendre, Pooja Singh, Swati Jaiswal

**Affiliations:** ^1^Department of Chemistry, Savitribai Phule Pune University (formerly known as Pune University), Pune, India; ^2^Pulmonary, Allergy and Critical Care Medicine, University of Alabama at Birmingham, Birmingham, AL, United States; ^3^Department of Medicine, UMass Chan Medical School, Worcester, MA, United States

**Keywords:** tuberculosis, extra-pulmonary tuberculosis, co-infections, molecular diagnosis, machine learning

Tuberculosis (TB) remains a persistent and formidable global health threat. According to the World Health Organization's most recent Global Tuberculosis Report, 10.8 million people developed TB in 2023, with 1.25 million lives lost to this preventable and curable disease (1). Despite decades of concerted efforts and the ambitious goals of the WHO's End TB Strategy, which seeks to reduce TB deaths by 90% and TB incidence by 80% by 2030 (2), the worldwide burden of TB remains alarmingly high.

The COVID-19 pandemic has further complicated TB control efforts, disrupting essential detection and treatment programs, and leading to increased morbidity and mortality (3, 4). Many of the gains achieved in TB control over the past decade have been eroded, and there is now a resurgence of TB in several high-burden regions. In this context, there is a renewed urgency to re-examine our strategies, foster innovation, and strengthen collaboration in TB research and care.

Our current Research Topic—*Tuberculosis: recent updates in basic research, drug discovery and treatment*, aims to promote both clinical and basic research focused on TB treatment, disease management, drug discovery, and how *Mycobacterium tuberculosis (M. tuberculosis)* adapts metabolically to different therapies. Key areas include understanding TB pathogenesis, developing new drugs and treatments, advancing vaccines and diagnostics, exploring innovative approaches for multidrug- and extensively drug-resistant TB, and investigating the molecular and genetic factors that influence mycobacterial responses to therapy, all to improve TB diagnosis, treatment, and global control (5). A comprehensive approach to TB must encompass not only the classic pulmonary form but also the growing complexity of extrapulmonary TB and its frequent intersection with other chronic conditions (6, 7). Deepening our understanding of TB pathogenesis -how *M. tuberculosis* infects, persists, and escapes immune surveillance—is fundamental to developing new therapeutic targets, more effective vaccines, and host-directed therapies (8–10). The dynamic interplay between bacterial virulence and host immunity largely determines whether TB remains latent or progresses to active disease (10, 11).

A pivotal study by He et al. employed bioinformatics and systems biology to investigate the biological links between chronic hepatitis B virus (HBV) infection and TB. The authors identified 35 shared genes that are involved in immune regulation and metabolism, revealing molecular pathways that may explain the increased risk of TB reactivation and adverse outcomes in individuals co-infected with HBV (He et al.). These findings highlight the need for integrated management strategies, given that immunosuppression or drug interactions can complicate the clinical course of both diseases. Adding to the understanding of host-pathogen interactions, Starshinova et al.'s review highlighted immune-neuroendocrine reactivity during pregnancy, a state that alters maternal immunity and increases vulnerability to TB. Pregnancy introduces unique immunological challenges for TB management. Hormonal changes during pregnancy shift the immune system toward an anti-inflammatory state, suppressing the T cell responses essential for TB control. This increases susceptibility to new TB infections and reactivation of latent disease, often resulting in atypical or subtle clinical presentations. Immune suppression peaks during labor and rebounds postpartum, influencing both TB progression and diagnostic reliability. These findings underscore the need for tailored screening and vigilant monitoring of pregnant women at risk for TB (Starshinova et al.). The complex interplay between TB and lung cancer was examined in a comprehensive review by Fang et al. Lung cancer patients are particularly susceptible to developing active TB, especially when treated with immunosuppressive agents such as immune checkpoint inhibitors (Fang et al.). The bidirectional relationship between TB and lung cancer is now well-recognized. Conversely, a history of TB increases the risk of lung cancer and is associated with poorer outcomes. Both diseases foster immunosuppressive microenvironments, characterized by regulatory T cells, myeloid-derived suppressor cells, and inhibitory cytokines, that undermine effective immune responses. Advances in spatial multi-omics are beginning to unravel these complex cellular interactions, paving the way for innovative therapies that target shared mechanisms of immune evasion and chronic inflammation.

An accurate and timely diagnosis is the cornerstone of effective TB control; however, this remains a significant challenge, particularly for extrapulmonary TB and in resource-limited settings. While chest X-rays (CXRs) have long been the primary method for pulmonary TB detection, their variable sensitivity and specificity often result in delayed or missed diagnoses. Fortunately, recent advances in technology are transforming TB diagnostics. Two articles featured in this Research Topic critically examined CXR limitations and highlighted emerging diagnostic modalities poised to address these gaps. Nanotechnology, for example, is revolutionizing the detection of bone and joint TB, which is notoriously difficult to diagnose early (Ding et al.). Palladium-platinum bimetallic nanoparticles (Pd@Pt NPs), when incorporated into paper-based diagnostic devices can now detect *M. tuberculosis* DNA in bone and joint samples within minutes, offering a rapid, sensitive, and portable solution for early diagnosis in even the most remote settings (Ding et al.). Another study by Guido et al. evaluated the comparative effectiveness of chest ultrasound, conventional chest X-ray, and AI-supported computer-aided diagnosis (CAD) for early TB detection in resource-limited environments, in the Ethiopian population, where the TB burden is highest. CXR limitations for pulmonary TB have also prompted the exploration of alternative imaging modalities and artificial intelligence-driven solutions (Guido et al.). Chest ultrasonography (CUS) provides a portable, radiation-free, and cost-effective alternative, while computer-aided diagnostic systems use machine learning to improve CXR interpretation accuracy and consistency.

In terms of diagnostic technology, Hu et al.'s meta-analysis of fluorescence in situ hybridization (FISH) demonstrated high sensitivity and specificity for pulmonary TB detection, especially in low-incidence settings. This suggests its potential role as a rapid, cost-effective diagnostic adjunct (Hu et al.). In addition to technological advances, TB is increasingly recognized as a complex, multisystem disease that often coexists with other chronic conditions. Zhou et al. reported a compelling case of a patient with both acute miliary pulmonary TB and immunoglobulin G4-related kidney disease (IgG4-RKD). The patient's overlapping clinical and laboratory features required a comprehensive diagnostic evaluation, but early recognition and individualized treatment led to full recovery—underscoring the importance of considering TB in the differential diagnosis of unexplained inflammatory or fibrotic diseases, especially in endemic areas.

## Advances in tuberculosis diagnostics and control

As we navigate the post-pandemic era, advancing tuberculosis diagnostics and control requires the integration of basic science, technological innovation, and clinical research. The convergence of TB with immune-mediated, viral, oncologic, and reproductive conditions calls for a more nuanced and multidisciplinary approach to care. Recent studies have highlighted progress in areas such as molecular co-infections, immune interactions, nanoparticle-based therapies, and AI-driven diagnostics—collectively paving the way for more effective and targeted interventions. Early recognition, personalized treatment, and collaborative research are essential to improving outcomes in high-risk populations. This Research Topic underscores the importance of sustained innovation and cross-disciplinary efforts to accelerate progress toward the WHO's End TB goals and address the evolving challenges of TB in an increasingly complex global health landscape.
